# *TNFSF4* Gene Variations Are Related to Early-Onset Autoimmune Thyroid Diseases and Hypothyroidism of Hashimoto’s Thyroiditis

**DOI:** 10.3390/ijms17081369

**Published:** 2016-08-20

**Authors:** Rong-Hua Song, Qiong Wang, Qiu-Ming Yao, Xiao-Qing Shao, Ling Li, Wen Wang, Xiao-Fei An, Qian Li, Jin-An Zhang

**Affiliations:** 1 Department of Endocrinology, Jinshan Hospital of Fudan University, No. 1508 Longhang Road, Jinshan District, Shanghai 201508, China; someonesrh66@163.com (R.-H.S.); 14211270001@fudan.edu.cn (Q.-M.Y.); shaoxq2015@163.com (X.-Q.S.); alana3344@126.com (L.L.); wwen910108@163.com (W.W.); anxiaofei2000@163.com (X.-F.A.); ellen38668@163.com (Q.L.); 2 The hemodialysis center of Nephropathy Department, Shaanxi Provincial People’s Hospital, No. 256 West Youyi Road, Beilin District, Xi’an 710068, China; wq9930@126.com

**Keywords:** tumor necrosis factor superfamily member 4 (*TNFSF4*), single nucleotide polymorphism (SNP), autoimmune thyroid diseases (AITDs), Graves’ disease (GD), Hashimoto’s thyroiditis (HT)

## Abstract

The aim of the current study was to examine whether the polymorphism loci of the tumor necrosis factor superfamily member 4 (*TNFSF4*) gene increase the risk of susceptibility to autoimmune thyroid diseases (AITDs) in the Han Chinese population, and a case-control study was performed in a set of 1,048 AITDs patients and 909 normal healthy controls in the study. A total of four tagging single nucleotide polymorphisms (SNPs) in the *TNFSF4* region, including rs7514229, rs1234313, rs16845607 and rs3850641, were genotyped using the method of ligase detection reaction. An association between GG genotype of rs3850641 in *TNFSF4* gene and AITDs was found (*p* = 0.046). Additionally, the clinical sub-phenotype analysis revealed a significant association between GG genotype in rs7514229 and AITDs patients who were ≤18 years of age. Furthermore, rs3850641 variant allele G was in strong association with hypothyroidism in Hashimoto’s thyroiditis (HT) (*p* = 0.018). The polymorphisms of the *TNFSF4* gene may contribute to the susceptibility to AITDs pathogenesis.

## 1. Introduction

Autoimmune thyroid diseases (AITDs) are a group of organ-specific and polygenic inherited autoimmune diseases, with an estimated prevalence of up to 1%–5% of the general population [1]. AITDs mainly consist of two clinical subtypes of Graves’ disease (GD) and Hashimoto’s thyroiditis (HT). GD is predominantly characterized by a variable combination of hyperthyroidism, diffused goiter and high level of thyroid stimulating hormone receptor antibody (TRAb). Meanwhile, some GD patients may present extrathyroidal manifestations, including ophthalmopathy, pretibial myxedema and clubbed fingers. Clinical features of HT include the presence of antibody against thyroid peroxidase (TPOAb) or thyroglobulin (TgAb). Additionally, some patients with HT harbor extensive apoptosis of thyrocytes leading to hypothyroidism. Although there are some common characteristics in GD and HT, such as destruction of thyroid tissue and the existence of circulating thyroid autoantibodies including TRAb, TPOAb and TgAb, the clinical presentations and mechanisms of the two subtypes are different from each other to some extent; for example, our previous studies found that GD, HT or even Graves’ ophthalmopathy (GO) have specific genetic backgrounds [2,3]. The pathogenesis of AITDs remains unclear, although there is much evidence demonstrating that the interaction between genetic factors and environmental components may be involved in their etiology [4,5].

More recently, an increasing body of research has confirmed that several specific genes are associated with multiple autoimmune diseases [6,7], implicating that many autoimmune diseases may share some genetic risk factors. For instance, TNFAIP3 has been identified to be related to the genetic etiology of systemic lupus erythematosus (SLE) [8], rheumatoid arthritis (RA) [9], systemic sclerosis (SSc) [10]. Additionally, we also found the relationship between this gene and GD [11]. All these data documented that variants in several genes probably contribute to dysregulation of common immune pathways, and then are involved in the pathological procedure of diverse autoimmune diseases.

The tumor necrosis factor superfamily member 4 (*TNFSF4*) gene encodes a cytokine (OX40L), which is expressed on antigen-presenting cells (APCs) to provide co-stimulatory signals to T cells. In recent years, the *TNFSF4* gene polymorphisms have been reported to be an important predisposition factor to SLE [12], RA [6], SSc [13] and primary Sjogren’s syndrome (pSS) [14]. However, to date, whether *TNFSF4* gene variations are associated with AITDs has not been investigated.

In the present study, we evaluated whether mutations in *TNFSF4* gene are genetically predisposed in Han Chinese populations to AITDs via a case-control study. Single nucleotide polymorphisms (SNPs) tagging four independent susceptibility loci were genotyped in a large cohort of AITDs patients and normal healthy controls. We also analyzed the association between each polymorphism locus and the predisposition to different subtypes of AITDs, including GD, HT and ophthalmopathy.

## 2. Results

### 2.1. Clinical Phenotype Analysis

The clinical characteristics of the AITDs cohort are displayed in Table 1. Among the investigated 1,048 AITDs patients, 693 were GD patients including 30.736% male and 69.264% female, with mean disease-onset age of 34.010 ± 14.395 years old; 355 were HT patients, including 12.676% male and 87.324% female, with mean disease-onset age of 32.720 ± 13.511 years old. There were 162 (15.458%) teenaged AITDs patients with disease-onset age ≤18 years old, 130 (12.405%) AITDs patients with ophthalmopathy, 198 (55.775%) HT patients with hypothyroidism, and 216 (20.611%) AITDs patients, comprised of 143 GD patients and 73 HT patients, with family history.

### 2.2. Allelic and Genotypic Association

There is a Hardy-Weinberg equilibrium (HWE) in the genotype distributions of *TNFSF4* SNPs in the control group (*p* > 0.05). Additionally, we evaluated the HWE for the loci in our AITDs cases; the HWE of the four loci for AITDs cases all had *p*-value higher than 0.01. In addition, variant genotype GG of rs3850641 in *TNFSF4* gene is associated with AITDs (*p* = 0.046), as shown in Table 2. Further analysis found that the frequencies of TT genotype in rs7514229 and GG genotype in rs3850641 were lower in the AITDs group than the control group (Table 3), which suggested that people with these genotypes are less susceptible to AITDs (*p* = 0.016, *OR* = 0.236, 95% CI = 0.066–0.850 and *p* = 0.027, *OR* = 0.492, 95% CI = 0.259–0.935, respectively). Those subjects whose genotypes of the four loci failed to be determined were ruled out from the statistical analysis. Moreover, the frequency of genotype GG in rs3850641 was lower in GD patients than the control group in analysis of sub-clinical types of AITDs (GD and HT) although without statistical significance (*p* = 0.086), as shown in Table 4.

### 2.3. Haplotypic Association

**Figure 1:**
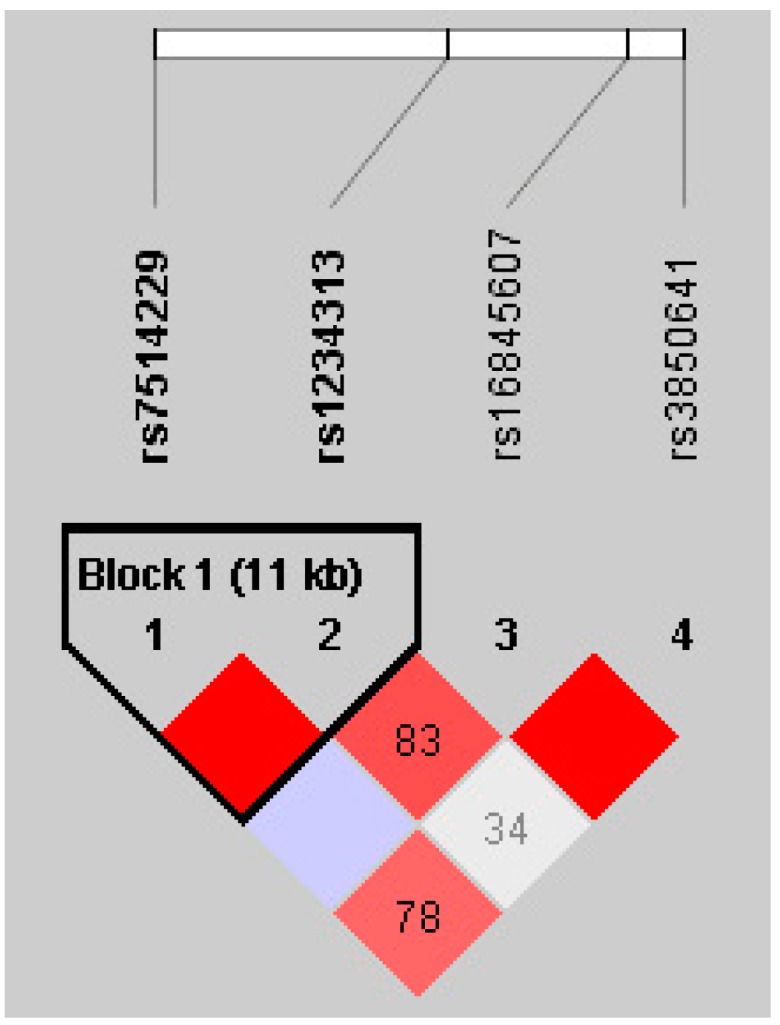
Linkage disequilibrium (LD) block of *TNFSF4* from controls in the Hapmap CHB data.

Haplotypic analysis using the Haploview software (Whitehead Institute for Biomedical Research, MIT Media Lab, and Broad Institute of Harvard and MIT, Cambridge, MA, USA) revealed that in the HapMap Han Chinese Beijing database, rs7514229 and rs1234313 were in the same block (Figure 1), which contained three haplotypes, namely GA, GG and TG. However, these haplotypes were not associated with AITDs (*p* > 0.05, data not shown).

### 2.4. Genotyping-Clinical Sub-Phenotype Association

To further investigate the relation between polymorphisms of *TNFSF4* and clinical phenotypes, clinical sub-phenotype analyses were conducted. The results showed that the frequency of genotype TT in rs7514229 marginally declined in AITDs patients with disease-onset age ≥19 years old (*p* = 0.049, as shown in Table 5). However, our present study displayed that *TNFSF4* gene variants were not associated with AITDs patients with ophthalmopathy or family history. Interestingly, the frequency of allele G in rs3850641 was significantly more decreased in HT patients with hypothyroidism than in HT patients without hypothyroidism, suggesting that HT patients with allele G in rs3850641 had increased susceptibility risk to hypothyroidism (*p* = 0.018, Table 6).

## 3. Discussion

The *TNFSF4* gene, also known as the OX40 ligand (OX40L), encodes the OX40L protein which is a co-stimulatory cytokine and belongs to the TNF ligand family. The protein mainly participates in the interaction of T-cell and antigen-presenting cell (APC), T-cell activation and B-cell differentiation, providing CD28-independent co-stimulatory signals for activated CD4^+^ T cells [15]. *TNFSF4*, located in chromosome 1 (1q25), contains three exons and two introns (in NCBI database). Previous studies have shown that polymorphisms of *TNFSF4* can confer risk to diverse autoimmune diseases, such as SLE, RA, SSc and pSS, but it remains unknown whether genetic mutations of *TNFSF4* region may induce occurrence of AITDs, which attracts our interest.

AITDs are also regarded as autoimmune diseases targeting the thyroid with a complex genetic and environmental etiology, manifesting mainly as GD and HT. It is notable that genetic factors play a prominent role in the occurrence and persistence of AITDs. Given that autoimmune diseases may share a common genetic predisposition, and that immune dysregulation plays a vital role in AITDs [16,17], we hypothesized that variants within the *TNFSF4* gene, which is a crucial immune regulator, could also elicit abnormal OX40L expression and dysfunction, thus affecting T-cell activation and leading to unbalanced immune regulation and its resultant occurrence of AITDs.

In the present work, we observed the association between four loci of *TNFSF4* gene and AITDs patients in the Han Chinese population. We found that the frequency of genotype GG in rs3850641 was slightly lower in AITDs patients, probably suggesting it could decrease susceptibility to AITDs. In addition, frequencies of GG genotype in rs3850641 and TT genotype in rs7514229 also decreased in AITDs subjects, confirming that variant genotype GG in rs3860541 was indeed a factor protecting people from AITDs, as was variant genotype TT in rs7514229. Our results suggested polymorphisms in the *TNFSF4* gene region, one SNP in 3′UTR (rs7514229) and two intronic SNPs (rs3860541 and rs1234313), may be associated with AITDs susceptibility. To our knowledge, variants in the intron of a gene may influence its expression and regulate its function [18], 3′UTR polymorphisms in the gene region are of important regulation function. We therefore speculated that the molecular action underlying genetic pathology of AITDs is that *TNFSF4* SNPs may affect the expression of *TNFSF4* gene and down-regulate T-cell activation, which requires further in-depth research to confirm.

Further, to investigate the association between genotype and clinical manifestations, we carried out the clinical sub-phenotype analysis. The AITDs occurrence in teenagers (≤18 young patients) may be due to their genetic family history of this disease [19,20], which corresponded with our results showing the frequency of family history was much higher in AITDs patients with disease-onset age ≤18 years old. Meanwhile, marginally significant differences in frequencies of rs7514229 genotype TT and disease-onset age were found between AITDs patients with disease-onset age ≤18 years old and AITDs patients with disease-onset age ≥19 years old. Similar correlations between gene mutations and disease-onset age were reported in RA [21], type 1 diabetes [22] and multiple sclerosis [23]. Furthermore, we revealed that frequency of GG genotype of rs3850641 declined slightly in GD subgroup of AITDs, although without significance. Nevertheless, we observed that *TNFSF4* SNPs were not associated with AITDs patients with ophthalmopathy or family history. Several studies provided clues that thyroid-associated ophthalmopathy (TAO) was correlated with the impact of environmental elements, especially current smoking history [24,25]. Recent studies are suggesting that genetic markers also affect the susceptibility of TAO [26], including genetic variants in the STAT3 [27], TSHR [28] and HLA-DR3 [29] regions. However, our results cannot add the *TNFSF4* gene to the list of the predisposition of thyroid-associated ophthalmopathy (TAO). Moreover, allele A from rs3850641 was associated with the decreased risk for the HT subgroup of hypothyroidism by 41.5%. In HT, hypothyroidism is more associated with a family history of thyroid dysfunction [20]. Our study showed HT hypothyroidism patients with higher ratio of family history, which was consistent with the previous research [20]. To our best knowledge, we were the first to find that genetic factors are also involved in etiology of hypothyroidism in HT. Why do these SNPs not show their susceptibility to GD or TAO? It is possible that thyroid eye disease or TAO is a different disease than Graves’ disease and Hashimoto’s thyroiditis. In addition, a recent paper found that polymorphisms in calsequestrin (CASQ1) are correlated with HT and GO, but not Graves’ hyperthyroiditis (GH) [30]. Interestingly, our study found SNPs in *TNFSF4* are associated with hypothyroidism of Hashimoto’s thyroiditis, but not thyroid orbitopathy or GD. These two studies do not show contradictory results, and illustrate the complexity of the diseases, GD, HT and TAO or GO. For instance, our previous studies indeed found *UBE2L3* and *CLEC16A* gene polymorphisms to be associated with susceptibility to HT rather than GD and TAO or GO [2,3]. Obviously, the genetic mechanisms of these diseases are still unclear, so more research is needed to reveal the pathomechanism of thyroid ophthalmopathy.

Overall, we provided the first evidence for genetic association between four susceptibility loci in the *TNFSF4* gene in Chinese AITDs patients, with samples exclusively from the Han Chinese population. Nevertheless, considering the validation of a convincing association and discovery of population differences, the importance of replication studies in some different populations should not be overlooked. The statistical power calculated in this research was very strong (larger than 0.8) to detect the association, and it has adequately reached a significant result. Simultaneously, the sample size in this study was large enough with 1,048 cases and 909 controls to effectively reduce the type of errors (type 1 error and type 2 error).

## 4. Materials and Methods

### 4.1. Subjects

A total of 1,048 Chinese patients with AITDs (693 GD and 355 HT) and 909 healthy Chinese controls were recruited. All AITDs patients were enrolled from the Out-Patient Department of Endocrinology of Jinshan Hospital of Fudan University. Ethnically and geographically matched and unrelated healthy controls were recruited from the Healthy Check-Up Center of the same hospital.

All AITDs patients were diagnosed as previously described [2,27]. GD patients were diagnosed based on their clinical manifestations and biochemical assessments of hyperthyroidism and the positive circulating TRAb, with or without positive TPOAb or TgAb and diffusive goiter of the thyroid. HT was defined based on the high level of either TPOAb or TgAb, with or without clinical and biochemical hypothyroidism and the presence of an enlarged thyroid. A minority of HT patients were further confirmed by fine needle aspiration biopsies. All the control subjects showed negative thyroid antibodies against TPO. In the current study, TPOAb, TgAb and TRAb were detected with highly specific and sensitive immunochemiluminescence kits from Roche Company (Shanghai, China).

All the subjects, including AITDs patients and controls, were ethnic Han Chinese. Written informed consent was obtained from all participants and the research was approved by the Ethics Committee of Jinshan Hospital of Fudan University (JYLL-2014-06, 2014/2/21), respectively.

### 4.2. DNA Sample Preparation

Genomic DNA were extracted from 2 mL of peripheral venous blood from each subject using RelaxGene Blood DNA System (Tiangen Biotech Company, Beijing, China), according to the manufacturer’s protocol. The concentration and A260/A280 ratio of all DNA samples were measured by NANO DROP 2000 Spectrophotometer (Thermo Scientific Company, Waltham, MA, USA). Finally, the DNA samples with great purity and concentration were used for next genotyping.

### 4.3. Single Nucleotide Polymorphism (SNP) Selection and Genotyping

Marker-tagging SNPs were chosen from the Hapmap CHB data using the Tagger programme of Haploview software (Whitehead Institute for Biomedical Research, MIT Media Lab, and Broad Institute of Harvard and MIT) to satisfy the following criteria: minor allele frequency (MAF) >0.1, Hardy-Weinberg equilibrium (HWE) with *p* > 0.001 and logarithm of odds (LOD) >3.0. For the *TNFSF4* gene of 23 kb with 42 SNPs in Hapmap CHB population, we selected four loci covering the whole region of the *TNFSF4* gene to capture all the most common variants. Four tag SNPs were selected including rs7514229 located in the 3′ untranslated region (UTR), as well as rs1234313, rs16845607 and rs3850641 in intron 1 of the *TNFSF4* region.

Genotyping of the four SNPs was undertaken using the ligase detection reaction (LDR) platform according to the manufacturer’s instructions. Moreover, to ensure detection quality, each reaction was performed in duplicate, and blank samples without DNA were used as negative controls. Furthermore, only SNPs and samples that passed the 95% quality control threshold were subjected to further statistical analysis and SNPs with allele frequencies not meeting Hardy-Weinberg equilibrium (HWE) were removed from the next analysis. The primers specific to the four SNPs at the *TNFSF4* loci are “rs7514229” forward-GATAACACAGAATCATCCAG and reverse-TTGTAGCACATGTTTCCCTG; “rs1234313” forward-ATCTAACACTGGCTCTAGTC and reverse-GCCATTCTGACTAGAATAGG; “rs16845607” forward-AGATATAGCTACCAAGCTCC and reverse-GATGAGAAAACAGAGGCTAC; “rs3850641” forward-GCTGTCACTTTGAAGCTTTG and reverse-TGCCTGATCAAACACATTAC.

### 4.4. Clinical Sub-Phenotype Analysis

Clinical sub-phenotype stratification analysis was conducted using a case-only approach, in which basic allelic and genotypic examination was performed by comparing minor allele and genotype frequency of cases with a specific sub-phenotype to the whole case group. The clinical sub-phenotypes include: (1) the age of disease onset (≤18 years old versus ≥19 years old); (2) presence or absence of ophthalmopathy which was defined as a distinctive disorder characterized by inflammation and swelling of the extraocular muscles, eyelid retraction, periorbital edema, episcleral vascular injection, conjunctive swelling and proptosis; (3) presence or absence of hypothyroidism in HT patients; and (4) presence or absence of AITDs family history, which was defined as the subjects’ first-degree relatives including parents, children and siblings or second-degree relatives such as grandparents, uncles and aunts who had AITDs.

### 4.5. Statistical Analysis

Clinical data were described as *M ± SD* (mean ± standard deviation). Hardy-Weinberg equilibrium (HWE) concordance test in the controls and patient samples, linkage disequilibrium (LD) test and haplotype frequency calculation were performed using HaploView 4.2 (Whitehead Institute for Biomedical Research, MIT Media Lab, and Broad Institute of Harvard and MIT). In order to analyze whether the four predisposing loci are associated with AITDs, allele and genotype frequencies were compared between AITDs cases and healthy controls using the Chi-square test (χ^2^-test) or Fisher’s exact test. LD among the selected SNPs was measured using the pairwise LD measures *D*’ and *r*^2^. All data were statistically calculated with the SPSS 18.0 software (International Business Machines Corporation, Armonk, NY, USA). A *p* value of less than 0.05 was considered statistically significant. Odds ratio (OR) and 95% confidence interval (95% CI) were applied to assess the association between each genotype and AITDs.

### 4.6. Power Calculation

Power calculations for AITDs in this research considered allele frequency of SNPs from 0.05 to 0.5, a population prevalence of 1%–5% for AITDs, and *OR* of 0.2–0.5 at a 0.05 significant level. As a result, this study had sufficient power (larger than 0.8) to detect the association of *OR* of 0.2 or above with 1,048 cases and 909 controls.

## 5. Conclusions

In conclusion, the preliminary findings of our present study are the first to indicate the association of novel genetic susceptibility loci of the *TNFSF4* region with the predisposition to AITDs. Additionally, our results support the importance of T cells in the pathology of AITDs, and reveal the frequency overlap of risk loci in immune pathways between AITDs and other autoimmune diseases.

## Figures and Tables

**Table 1:** Clinical data of AITDs patients and controls.

Clinical Phenotype	AITDs (%)	GD (%)	HT (%)	Control (%)
Number	1048	693	355	909
Gender	–	–	–	–
Male	260 (24.809)	213 (30.736)	45 (12.676)	314 (34.543)
Female	788 (75.191)	480 (69.264)	310 (87.324)	595 (65.457)
Onset of age	33.570 ± 14.108	34.010 ± 14.395	32.720 ± 13.511	–
≤18 years	162 (15.458)	113 (16.306)	49 (13.803)	–
≥19 years	886 (84.542)	580 (83.694)	306 (86.197)	–
Ophthalmopathy	–	–	–	–
(+)	130 (12.405)	124 (17.893)	6 (1.690)	–
(−)	918 (87.595)	569 (82.107)	349 (98.310)	–
Family history	–	–	–	–
(+)	216 (20.611)	143 (20.635)	73 (20.563)	–
(−)	832 (79.389)	550 (79.365)	282 (79.437)	–

**Table 2:** Allele and genotype distribution of *TNFSF4* in AITDs patients and controls.

SNP ID	Control	AITDs	*p*	*OR* (95% CI)
rs7514229	–	–	–	–
GG	732 (81.424)	838 (81.518)	0.052	–
TT	11 (1.224)	3 (0.292)	–	–
GT	156 (17.353)	187 (18.191)	–	–
G	1620 (90.100)	1863 (90.613)	0.590	1.061 (0.856–1.314)
T	178 (9.900)	193 (9.387)	–	–
rs1234313	–	–	–	–
AA	365 (40.466)	445 (42.871)	0.536	–
GG	110 (12.195)	117 (11.272)	–	–
AG	427 (47.339)	476 (45.857)	–	–
A	1157 (64.135)	1366 (65.800)	0.278	1.076 (0.943–1.228)
G	647 (35.865)	710 (34.200)	–	–
rs16845607	–	–	–	–
AA	4 (0.443)	5 (0.480)	0.993	–
GG	797 (88.359)	920 (88.292)	–	–
AG	101 (11.197)	117 (11.228)	–	–
*A*	109 (6.042)	127 (6.094)	0.946	1.009 (0.775–1.314)
*G*	1695 (93.958)	1957 (93.906)	–	–
rs3850641	–	–	–	–
*GG*	26 (2.879)	15 (1.438)	0.046	–
*AA*	660 (73.090)	750 (71.908)	–	–
*AG*	217 (24.031)	278 (26.654)	–	–
*G*	269 (14.895)	308 (14.765)	0.910	0.990 (0.829–1.182)
*A*	1537 (85.105)	1778 (85.235)	–	–

**Table 3:** Genotype frequency of *TNFSF4* loci in AITDs patients and controls.

SNP Name	Genotype	Control (%)	AITDs (%)	*p*	*OR*	95% CI
rs7514229	GG	732 (81.424)	838 (81.518)	0.958	1.006	0.799–1.267
	TT + GT	167 (18.576)	190 (18.482)			
	TT	11 (1.233)	3 (0.292)	0.016	0.236	0.066–0.850
	GG + GT	888 (98.776)	1025 (99.708)			
	GT	156 (17.353)	187 (18.191)	0.631	1.059	0.838–1.339
	GG + TT	743 (82.647)	841 (81.809)			
rs1234313	AA	365 (40.466)	445 (42.871)	0.284	1.104	0.921–1.323
	GG + AG	537 (59.534)	593 (57.129)			
	GG	110 (12.195)	117 (11.272)	0.528	0.915	0.693–1.206
	AA + AG	792 (87.805)	921 (88.728)			
	AG	427 (47.339)	476 (45.857)	0.514	0.943	0.788–1.126
	AA + GG	475 (52.661)	562 (54.143)			
rs16845607	AA	4 (0.443)	5 (0.480)	0.906	1.082	0.290–4.049
	GG+AG	898 (99.557)	1037 (99.520)			
	GG	797 (88.359)	920 (88.292)	0.963	0.993	0.752–1.311
	AA + AG	105 (11.641)	122 (11.708)			
	AG	101 (11.197)	117 (11.228)	0.983	1.003	0.756–1.330
	AA + GG	801 (88.803)	925 (88.772)			
rs3850641	GG	26 (2.879)	15 (1.438)	0.027	0.492	0.259–0.935
	AA + AG	877 (97.121)	1028 (98.562)			
	AA	660 (73.090)	750 (71.908)	0.561	0.943	0.772–1.151
	GG + AG	243 (26.910)	293 (28.092)			
	AG	217 (24.031)	278 (26.654)	0.185	1.149	0.935–1.410
	AA + GG	686 (75.969)	765 (73.346)			

**Table 4:** Distribution of genotype and allele of *TNFSF4* gene in sub-clinical types of AITDs patients and controls.

SNP	Control	GD	*p*	*OR* (95% CI)	HT	*p*	*OR* (95% CI)
rs7514229	–	–	–	–	–	–	–
GG	732 (81.424)	560 (82.474)	0.128	–	278 (79.656)	0.182	–
TT	11 (1.224)	2 (0.295)	–	–	1 (0.287)	–	–
GT	156 (17.353)	117 (17.231)	–	–	70 (20.057)	–	–
G	1620 (90.100)	1237 (91.090)	0.347	1.123 (0.881–1.432)	626 (89.685)	0.756	0.955 (0.716–1.275)
T	178 (9.900)	121 (8.910)	–	–	72 (10.315)	–	–
rs1234313	–	–	–	–	–	–	–
AA	365 (40.466)	298 (43.504)	0.474	–	147 (41.643)	0.832	–
GG	110 (12.195)	78 (11.387)	–	–	39 (11.048)	–	–
AG	427 (47.339)	309 (45.109)	–	–	167 (47.309)	–	–
A	1157 (64.135)	905 (66.058)	0.261	1.088 (0.939–1.261)	461 (65.297)	0.584	1.052 (0.877–1.263)
G	647 (35.865)	465 (33.942)	–	–	245 (34.703)	–	–
rs16845607	–	–	–	–	–	–	–
AA	4 (0.443)	4 (0.581)	0.897	–	1 (0.283)	0.859	–
GG	797 (88.359)	605 (87.808)	–	–	315 (89.235)	–	–
AG	101 (11.197)	80 (11.611)	–	–	37 (10.482)	–	–
*A*	109 (6.042)	88 (6.386)	0.69	1.061(0.794–1.418)	39 (5.524)	0.62	0.909 (0.624–1.325)
*G*	1695 (93.958)	1290 (93.614)	–	–	667 (94.476)	–	–
rs3850641	–	–	–	–	–	–	–
GG	26 (2.879)	9 (1.306)	0.086	–	6 (1.695)	0.172	–
AA	660 (73.090)	502 (72.859)	–	–	248 (70.056)	–	–
AG	217 (24.031)	178 (25.835)	–	–	100 (28.249)	–	–
G	269 (14.895)	196 (14.224)	0.595	0.948(0.776–1.156)	112 (15.819)	0.561	1.074 (0.845–1.364)
A	1537 (85.105)	1182 (85.776)	–	–	596 (84.181)	–	–

**Table 5:** Allele and genotype distribution of *TNFSF4* in AITDs patients with or without early-onset age.

SNP ID	Onset Age of AITDs Patients		*p*	*OR* (95% CI)
	≤18	≥19		
rs7514229	–	–	–	–
GG	128 (80.503)	710 (81.703)	0.049	–
TT	2 (1.258)	1 (0.115)	–	–
GT	29 (18.239)	158 (18.182)	–	–
G	285 (89.623)	1578 (90.794)	0.51	1.142 (0.769–1.696)
T	33 (10.377)	160 (9.206)	–	–
rs1234313	–	–	–	–
AA	71 (44.375)	374 (42.597)	0.41	–
GG	22 (13.750)	95 (10.820)	–	–
AG	67 (41.875)	409 (46.583)	–	–
A	209 (65.312)	1157 (65.888)	0.842	1.026 (0.799–1.318)
G	111 (34.688)	599 (34.112)	–	–
rs16845607	–	–	–	–
AA	1 (0.625)	4 (0.454)	0.246	–
GG	135 (84.375)	785 (89.002)	–	–
AG	24 (15.000)	93 (10.544)	–	–
A	26 (8.125)	101 (5.726)	0.099	0.687 (0.439–1.075)
G	294 (91.875)	1663 (94.274)	–	–
rs3850641	–	–	–	–
GG	2 (1.250)	13 (1.472)	0.639	–
AA	120 (75.000)	630 (71.348)	–	–
AG	38 (23.750)	240 (27.180)	–	–
G	42 (13.125)	266 (15.062)	0.369	1.174 (0.827–1.664)
A	278 (86.875)	1500 (84.938)	–	–

**Table 6:** *TNFSF4* genotype and allele distribution in clinical sub-phenotype of HT patients.

*TNFSF4* SNP	HT		*p*	*OR* (95% CI)
	Non-Hypothyroidism	Hypothyroidism		
rs7514229	–	–	–	–
GG	107 (80.451)	153 (78.462)	0.668	–
TT	0 (0)	1 (0.513)	–	–
GT	26 (19.549)	41 (21.026)	–	–
G	240 (90.226)	347 (88.974)	0.608	0.874 (0.523–1.462)
T	26 (9.774)	43 (11.026)	–	–
rs1234313	–	–	–	–
AA	50 (37.037)	90 (45.685)	0.287	
GG	16 (11.852)	19 (9.645)	–	–
AG	69 (51.111)	88 (44.670)	–	–
A	169 (62.593)	268 (68.020)	0.148	1.271 (0.918–1.759)
G	101 (37.407)	126 (31.980)	–	–
rs16845607	–	–	–	–
AA	1 (0.741)	0 (0)	0.136	
GG	115 (85.185)	180 (91.371)	–	–
AG	19 (14.074)	17 (8.629)	–	–
A	21 (7.778)	17 (4.315)	0.059	0.535 (0.277–1.034)
G	249 (92.22)	377 (95.685)	–	–
rs3850641	–	–	–	–
GG	1 (0.741)	5 (2.525)	0.051	
AA	104 (77.037)	129 (65.152)	–	–
AG	30 (22.222)	64 (32.323)	–	–
G	32 (11.852)	74 (18.687)	0.018	1.709 (1.096–2.674)
A	238 (88.148)	322 (81.313)	–	–

## References

[1] Tomer Y. (2014). Mechanisms of autoimmune thyroid diseases: From genetics to epigenetics. Annu. Rev. Pathol..

[2] Muhali F.S., Cai T.T., Zhu J.L., Qin Q., Xu J., He S.T., Shi X.H., Jiang W.J., Xiao L., Li D.F. (2014). Polymorphisms of CLEC16A region and autoimmune thyroid diseases. G3 (Bethesda).

[3] Wang Y., Zhu Y.F., Wang Q., Xu J., Yan N., Xu J., Shi L.F., He S.T., Zhang J.A. (2016). The haplotype of *UBE2L3* gene is associated with Hashimoto’s thyroiditis in a Chinese Han population. BMC Endocr. Disord..

[4] Balazs C. (2012). The role of hereditary and environmental factors in autoimmune thyroid diseases. Orv. Hetil..

[5] Effraimidis G., Wiersinga W.M. (2014). Mechanisms in endocrinology: Autoimmune thyroid disease: Old and new players. Eur. J. Endocrinol..

[6] Orozco G., Eyre S., Hinks A., Bowes J., Morgan A.W., Wilson A.G., Wordsworth P., Steer S., Hocking L., Thomson W. (2011). Study of the common genetic background for rheumatoid arthritis and systemic lupus erythematosus. Ann. Rheum. Dis..

[7] Gourh P., Arnett F.C., Tan F.K., Assassi S., Divecha D., Paz G., McNearney T., Draeger H., Reveille J.D., Mayes M.D. (2010). Association of TNFSF4 (OX40L) polymorphisms with susceptibility to systemic sclerosis. Ann. Rheum. Dis..

[8] Graham R.R., Cotsapas C., Davies L., Hackett R., Lessard C.J., Leon J.M., Burtt N.P., Guiducci C., Parkin M., Gates C. (2008). Genetic variants near TNFAIP3 on 6q23 are associated with systemic lupus erythematosus. Nat. Genet..

[9] Bowes J., Lawrence R., Eyre S., Panoutsopoulou K., Orozco G., Elliott K.S., Ke X., Morris A.P., Thomson W., Worthington J. (2010). Rare variation at the TNFAIP3 locus and susceptibility to rheumatoid arthritis. Hum. Genet..

[10] Dieude P., Guedj M., Wipff J., Ruiz B., Riemekasten G., Matucci-Cerinic M., Melchers I., Hachulla E., Airo P., Diot E. (2010). Association of the TNFAIP3 rs5029939 variant with systemic sclerosis in the european caucasian population. Ann. Rheum. Dis..

[11] Song R.H., Yu Z.Y., Wang Q., Muhali F.S., Jiang W.J., Xiao L., Shi X.H., He S.T., Xu J., Zhang J.A. (2014). Polymorphisms of the TNFAIP3 region and Graves’ disease. Autoimmunity.

[12] Zhou X.J., Lu X.L., Nath S.K., Lv J.C., Zhu S.N., Yang H.Z., Qin L.X., Zhao M.H., Su Y., Shen N. (2012). Gene–gene interaction of BLK, TNFSF4, TRAF1, TNFAIP3, and REL in systemic lupus erythematosus. Arthritis Rheum..

[13] Coustet B., Bouaziz M., Dieude P., Guedj M., Bossini-Castillo L., Agarwal S., Radstake T., Martin J., Gourh P., Elhai M. (2012). Independent replication and meta analysis of association studies establish TNFSF4 as a susceptibility gene preferentially associated with the subset of anticentromere-positive patients with systemic sclerosis. J. Rheumatol..

[14] Sun F., Li P., Chen H., Wu Z., Xu J., Shen M., Leng X., Shi Q., Zhang W., Tian X. (2013). Association studies of TNFSF4, TNFAIP3 and FAM167A-BLK polymorphisms with primary Sjogren’s syndrome in Han Chinese. J. Hum. Genet..

[15] Kinnear G., Wood K.J., Fallah-Arani F., Jones N.D. (2013). A diametric role for OX40 in the response of effector/memory CD4^+^ T cells and regulatory T cells to alloantigen. J. Immunol..

[16] Simmonds M.J. (2013). GWAS in autoimmune thyroid disease: Redefining our understanding of pathogenesis. Nat. Rev. Endocrinol..

[17] Marique L., Van Regemorter V., Gerard A.C., Craps J., Senou M., Marbaix E., Rahier J., Daumerie C., Mourad M., Lengele B. (2014). The expression of dual oxidase, thyroid peroxidase, and caveolin-1 differs according to the type of immune response (Th1/Th2) involved in thyroid autoimmune disorders. J. Clin. Endocrinol. Metab..

[18] Alberobello A.T., Congedo V., Liu H., Cochran C., Skarulis M.C., Forrest D., Celi F.S. (2011). An intronic SNP in the thyroid hormone receptor β gene is associated with pituitary cell-specific over-expression of a mutant thyroid hormone receptor β2 (R338W) in the index case of pituitary-selective resistance to thyroid hormone. J. Transl. Med..

[19] Brix T.H., Petersen H.C., Iachine I., Hegedus L. (2003). Preliminary evidence of genetic anticipation in Graves’ disease. Thyroid.

[20] Manji N., Carr-Smith J.D., Boelaert K., Allahabadia A., Armitage M., Chatterjee V.K., Lazarus J.H., Pearce S.H., Vaidya B., Gough S.C. (2006). Influences of age, gender, smoking, and family history on autoimmune thyroid disease phenotype. J. Clin. Endocrinol. Metab..

[21] Radstake T.R., Barrera P., Albers M.J., Swinkels H.L., van de Putte L.B., van Riel P.L. (2001). Genetic anticipation in rheumatoid arthritis in Europe. European consortium on rheumatoid arthritis families. J. Rheumatol..

[22] Paterson A.D., Kennedy J.L., Petronis A. (1996). Evidence for genetic anticipation in non-mendelian diseases. Am. J. Hum. Genet..

[23] Cocco E., Sardu C., Lai M., Spinicci G., Contu P., Marrosu M.G. (2004). Anticipation of age at onset in multiple sclerosis: A sardinian cohort study. Neurology.

[24] Villanueva R., Inzerillo A.M., Tomer Y., Barbesino G., Meltzer M., Concepcion E.S., Greenberg D.A., MacLaren N., Sun Z.S., Zhang D.M. (2000). Limited genetic susceptibility to severe Graves’ ophthalmopathy: No role for CTLA-4 but evidence for an environmental etiology. Thyroid.

[25] Melcescu E., Horton W.B., Kim D., Vijayakumar V., Corbett J.J., Crowder K.W., Pitman K.T., Uwaifo G.I., Koch C.A. (2014). Graves orbitopathy: Update on diagnosis and therapy. South. Med. J..

[26] Li H., Chen Q. (2013). Genetic susceptibility to Grave’s disease. Front. Biosci..

[27] Xiao L., Muhali F.S., Cai T.T., Song R.H., Hu R., Shi X.H., Jiang W.J., Li D.F., He S.T., Xu J. (2013). Association of single-nucleotide polymorphisms in the *STAT3* gene with autoimmune thyroid disease in chinese individuals. Funct. Integr. Genom..

[28] Liu L., Wu H.Q., Wang Q., Zhu Y.F., Zhang W., Guan L.J., Zhang J.A. (2012). Association between thyroid stimulating hormone receptor gene intron polymorphisms and autoimmune thyroid disease in a Chinese Han population. Endocr. J..

[29] Jurecka-Lubieniecka B., Ploski R., Kula D., Krol A., Bednarczuk T., Kolosza Z., Tukiendorf A., Szpak-Ulczok S., Stanjek-Cichoracka A., Polanska J. (2013). Association between age at diagnosis of Graves’ disease and variants in genes involved in immune response. PLoS ONE.

[30] Lahooti H., Cultrone D., Edirimanne S., Walsh J.P., Delbridge L., Cregan P., Champion B., Wall J.R. (2015). Novel single-nucleotide polymorphisms in the calsequestrin-1 gene are associated with Graves’ ophthalmopathy and Hashimoto’s thyroiditis. Clin. Ophthalmol..

